# Twisted orientation of the muscle bundles in the levator ani functional parts in women: Implications for pelvic floor support mechanism

**DOI:** 10.1111/joa.13968

**Published:** 2023-10-26

**Authors:** Satoru Muro, Shoko Moue, Keiichi Akita

**Affiliations:** ^1^ Department of Clinical Anatomy Tokyo Medical and Dental University Tokyo Japan

**Keywords:** anatomy, defaecation, levator ani, pelvic floor, perineum, urination

## Abstract

This study presents a comprehensive investigation of the anatomical features of the levator ani muscle. The levator ani is a critical component of the pelvic floor; however, its intricate anatomy and functionality are poorly understood. Understanding the precise anatomy of the levator ani is crucial for the accurate diagnosis and effective treatment of pelvic floor disorders. Previous studies have been limited by the lack of comprehensive three‐dimensional analyses; to overcome this limitation, we analysed the levator ani muscle using a novel 3D digitised muscle‐mapping approach based on layer‐by‐layer dissection. From this examination, we determined that the levator ani consists of overlapping muscle bundles with varying orientations, particularly in the anteroinferior portion. Our findings revealed distinct muscle bundles directly attached to the rectum (LA‐re) and twisted muscle slings surrounding the anterior (LA‐a) and posterior (LA‐p) aspects of the rectum, which are considered functional parts of the levator ani. These results suggest that these specific muscle bundles of the levator ani are primarily responsible for functional performance. The levator ani plays a crucial role in rectal elevation, lifting the centre of the perineum and narrowing the levator hiatus. The comprehensive anatomical information provided by our study will enhance diagnosis accuracy and facilitate the development of targeted treatment strategies for pelvic floor disorders in clinical practice.

## Graphical abstract

This study explores the intricate anatomy of the levator ani muscle, a key component of the pelvic floor. Using a novel three‐dimensional digitised muscle mapping, the study clarifies specific muscle bundles directly attached to the rectum and twisted muscle slings surrounding its anterior and posterior aspects. These findings enhance diagnostic accuracy and enable the development of targeted treatment strategies for pelvic floor disorders.

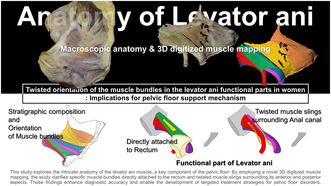

## 1 INTRODUCTION

Pelvic floor dysfunction is a common problem among women and can result in various symptoms, including urinary incontinence, faecal incontinence, constipation, dyspareunia and pelvic pain or pressure (Lawson & Sacks, 2018; Quaghebeur et al., 2021). This condition occurs when the muscles and connective tissues supporting pelvic organs become weakened or damaged (Bø, 2004; Doxford‐Hook et al., 2022). In particular, pelvic organ prolapse is strongly associated with injury to the levator ani (LA) (DeLancey et al., 2003; Lien et al., 2004; Miller et al., 2010, 2015). The LA is the largest muscle that provides support to the pelvic organs and, in tandem with the coccygeus, constitutes the pelvic diaphragm (pelvic floor muscle) (Muro & Akita, 2023b). LA function is indispensable for pelvic floor support and is closely related to pathologies such as urinary incontinence and pelvic organ prolapse in women; therefore, its anatomy has received much attention.

LA anatomy is generally described by three subdivisions: pubococcygeus, puborectalis and iliococcygeus (Standring & Gray, 2015). The pubococcygeus originates from the pubis and mainly inserts into the pelvic organs (viscera), and another name, *pubovisceralis*, was proposed by Lawson (1974), which was later adopted by Kearney et al. (2004). The puborectalis muscle originates from the pubis and surrounds the posterior rectum in a sling‐like manner. The iliococcygeus originates from the tendinous arch of the levator ani (TALA) and is inserted into the coccyx. While these subdivisions are distinct, the entire LA closes the pelvic outlet and is generally seen as a single sheet‐like structure. However, there are few detailed reports on the stratigraphic composition of the LA and orientation of the muscle bundles. It remains unclear which part of the LA originates from the pubic bone, which part originates from the TALA and the correspondence between each origin and insertion.

We have reported several structures for the insertion of the LA directly involved in the rectum: the skeletal muscle fibres of the LA innermost muscle bundle are directly attached to the smooth muscle fibres of the longitudinal muscles of the rectum (Muro et al., 2018; Tsukada et al., 2016); the LA includes muscle bundles surrounding both the rectum anterior and posterior (Baramee et al., 2020; Suriyut et al., 2020). Based on these findings, the part of the LA that is structurally directly involved in the rectum consists of muscle bundles directly attached to the rectum and muscle bundles surrounding the rectum anteriorly and posteriorly. Focusing on these muscle bundles, we hypothesised that these parts of the LA may not be simple single sheets, but may have a complex layered structure and different muscle bundle orientations from other parts. Once the detailed stratified relationship of the LA muscle bundles is clarified, it is possible to identify the functional part of the LA based on its structural characteristics. A detailed anatomical understanding of the functional part of the LA will contribute to understanding the pelvic floor support mechanism and pathophysiology of pelvic organ prolapse and urinary incontinence, as well as provide a basis for the development of optimal treatment strategies. The aim of this study was to comprehensively analyse and provide detailed photographic documentation of dissections of the muscle bundles of the LA in adult specimens to clarify the correspondence between the origin and insertion and the stratified relationship of the muscle bundles.

## 2 MATERIALS AND METHODS

### 2.1 Preparation of cadaveric specimens

All the cadavers used in this study were donated to our department. The donation document format was congruent with the Japanese law entitled ‘The Act on Body Donation for Medical and Dental Education’ (Act No. 56 of 1983). All donors voluntarily declared before their passing that their bodies could be donated for use as educational and research materials. Informed consent was obtained after explaining the purpose and methods for using corpses from body donors. After they passed away, we explained their informed consent to their relatives and confirmed that there was no opposition. All cadavers were fixed using arterial perfusion with 8% formalin and preserved in 30% ethanol. The study was approved by the Board of Ethics of the Tokyo Medical and Dental University (approval number: M2018‐006). All methods were performed according to the relevant guidelines and regulations.

### 2.2 Macroscopic anatomy

Thirty‐one female cadavers (mean age at death, 86.6 years; age range, 49–103 years) were used for the macroscopic anatomy. Cadavers with pathologies or abnormalities affecting the pelvic floor musculature were excluded. Among them, 21 cadavers were used for (1) the layer‐by‐layer dissection, eight for (2) the whole‐mount dissection and two for (3) the muscle isolation method. Initially, the pelvic region was obtained from cadavers, whereby the superior margin of the pelvic specimen was transected across the abdomen at the level of the iliac crest, and the lower portion was resected at the hip joint. Subsequently, for the specimens used for layer‐by‐layer dissection and muscle isolation, the pelvis was sectioned in the median plane using a diamond band pathology saw (EXAKT 312; EXAKT Advanced Technologies, Norderstedt, Germany). A total of 38 pelvic halves from 21 cadavers were used for layer‐by‐layer dissection, and three pelvic halves from two cadavers were used for muscle isolation.

1. Layer‐by‐layer dissection

During layer‐by‐layer dissection, the pelvic halves specimens were dissected from the medial aspect. This approach has been employed in our previous studies and allows for the sequential demonstration of layered structures while maintaining the spatial positioning of the rectum and pelvic muscles (Muro et al., 2014, 2018, 2019, 2020, 2023). In the present investigation, following the observation of the structures in the median section, the LA and coccygeus were dissected while preserving the rectum, and the attachment site of the LA to the rectum was examined. Thereafter, the rectum was removed to reveal the muscle bundles of the LA, which were subsequently dissected along the stratigraphic sequence, allowing for the observation of all muscle bundles comprising the LA from the medial aspect.

2 Whole‐mount dissection

In whole‐mount dissection, the posterior portion of the pelvis, including the sacrum, was excised, while the anterior portions of the acetabulum and rectum were preserved, and the muscles of the LA and coccygeus were dissected. The LA was visualised from the superior and anterior aspects, enabling observation of its attachment to the rectum and the orientation of its muscle bundles.

3 Muscle isolation method

The muscle isolation method allows for the detailed observation of muscle bundles and tendon properties. Initially, the pelvic halves were dissected from the medial aspect to expose the LA, coccygeus and external anal sphincter. These muscles were then isolated by detachment from their attachment *en bloc* and degreased with a 30% ethanol soak; connective tissue and fat were removed to expose the muscle bundles, tendons and aponeuroses. Subsequently, isolated muscle specimens were viewed from both the medial and lateral sides to observe the orientation of the muscle bundles.

### 2.3 3D digitised muscle mapping

To visualise the spatial positional relationship between muscle bundles, each step of the layer‐by‐layer dissection process involved in the sequential removal of structures according to their stratigraphy was scanned in 3D (EinScan‐SE; Japan 3D Printer Co., Ltd) (Muro et al., 2023). The acquired 3D data were aligned and processed using Autodesk Meshmixer version 3.1 (Autodesk; https://www.meshmixer.com/). The LA and coccygeus were then separated and superimposed on a digital 3D space to reconstruct the muscles. The constructed digital 3D data were used to analyse the layer composition and muscle bundles of the LA using MeshLab version 2022.02 (ISTICNR; https://www.meshlab.net/) (Cignoni et al., 2008; Muro & Akita, 2023a). Finally, the 3D data were exported to a 3D PDF format using the PDF3D ReportGen version 2.22.1.11324 (Visual Technology Services Ltd; https://www.pdf3d.com/; VTS Software; https://vts‐software.co.jp/) to facilitate data sharing (see Supporting Information S1).

In this article, considering the current terminology confusion, we decided not to use the general anatomical names of the LA subdivisions (pubococcygeus, puborectalis and iliococcygeus), and instead describe the structures solely based on muscle bundle composition and insertion.

## 3 RESULTS

### 3.1 Stratified relationship of muscle bundles

**FIGURE 1:**
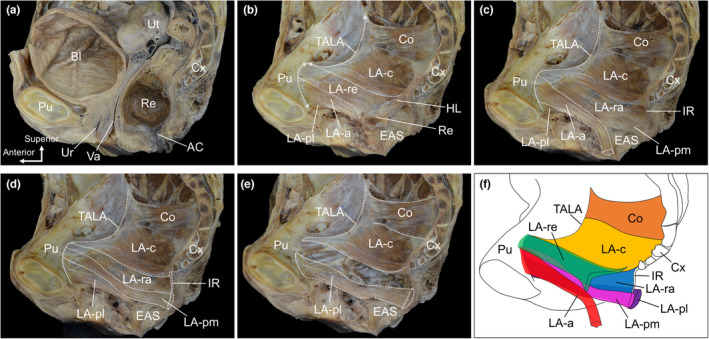
Stratified relationship of the levator ani muscle bundles. (a) Medial aspect of the female pelvis after sectioning in the median plane. (b) After removing most of the pelvic organs. The anteroinferior and posterosuperior components of the LA originate from the pubis (double‐headed arrow line) and TALA (double‐headed dashed arrow line) respectively. The LA‐re was attached to the rectum. (c) After removal of the LA‐re. The LA‐a surrounds the anterior side of the rectum. (d) After removal of the LA‐a. The LA‐pm surrounds the posterior rectum. The LA‐ra formed the IR. (e) After removal of the LA‐pm and LA‐ra. The LA‐pl laterally surrounded the posterior side of the rectum. The LA‐c then inserted into the coccyx. (f) Schematic of the stratified relationship of LA muscle bundles. The posterosuperior part of the LA was observed as a thin single sheet (LA‐ra and LA‐c), whereas the anteroinferior part of the LA had a complex layered configuration with overlapping muscle bundles (LA‐re, LA‐a, LA‐pm and LA‐pl). AC, anal canal; Bl, bladder; Co, coccygeus; Cx, coccyx; EAS, external anal sphincter; HL, hiatal ligament; IR, iliococcygeal raphe; LA, levator ani; LA‐a, LA muscle bundles surrounding anterior side of the Re; LA‐c, LA muscle bundles attaching to the coccyx; LA‐pl, LA muscle bundles laterally surrounding the posterior side of the Re; LA‐pm, LA muscle bundles medially surrounding the posterior side of the Re; LA‐ra, LA muscle bundles attaching to IR; LA‐re, LA muscle bundles attaching to the Re; Pu, pubis; Re, rectum; TALA, tendinous arch of levator ani; Ur, urethra; Ut, uterus; Va, vagina.

The pelvic organs were observed in the median sections of the bladder, urethra, uterus, vagina, rectum and anal canal (Figure 1a). After removing most of the pelvic organs, only the portion of the rectal wall attached to the LA was maintained and the medial aspect of the LA was dissected (Figure 1b). The LA was observed to be a broad muscle that enveloped the pelvic wall, with the anteroinferior component originating from the pubis (indicated by the double‐headed arrow in Figure 1b) and the posterosuperior component originating from the TALA (indicated by the double‐headed dashed arrow in Figure 1b). The coccygeus was situated superior to the LA, and the external anal sphincter was situated inferiorly. The innermost LA muscle bundles were attached to the rectum (LA‐re) (Figure 1b). When the LA‐re was removed together with the rectal wall, the muscle bundles located inferolateral to the LA‐re were observed to surround the anterior side of the rectum (LA‐a) (Figure 1c). After removal of the LA‐a, muscle bundles located superolateral to it were found, including muscle bundles surrounding the posterior side of the rectum (LA‐pm) and muscle bundles forming the iliococcygeal raphe below the coccyx (LA‐ra) (Figure 1d). When the LA‐pm and LA‐ra were removed, the muscle bundles surrounding the posterior side of the rectum were located lateral to them (LA‐pl) (Figure 1e). The outermost and uppermost muscle bundles of the LA are inserted into the coccyx (LA‐c). Above the LA‐c was the coccygeus. The LA‐re, LA‐a, LA‐pm and LA‐pl originated from the pubis. Some muscle bundles of the LA‐ra were found to originate from the pubis, whereas others originated from the TALA. LA‐c originates from TALA. Overall, the posterosuperior part of the LA was observed as a thin single sheet (LA‐ra and LA‐c), whereas the anteroinferior part had a complex layered configuration with overlapping muscle bundles (LA‐re, LA‐a, LA‐pm and LA‐pl) (Figure 1f). This complicated anteroinferior part included muscle bundles attached to the rectum (LA‐re) and muscle bundles surrounding the rectum anteriorly and posteriorly (LA‐a, LA‐pm and LA‐pl), which were directly structurally associated with the rectum.

**FIGURE 2:**
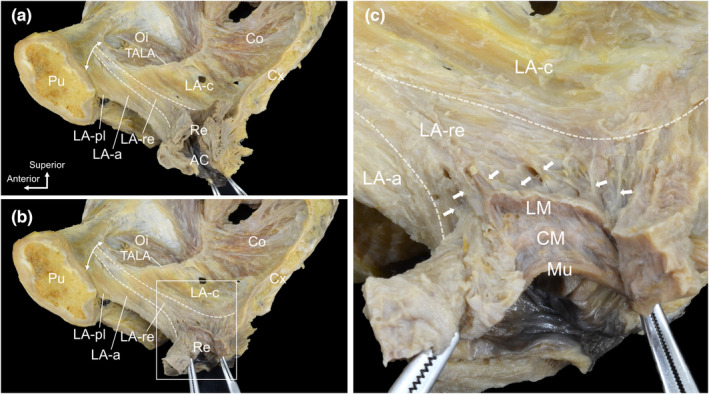
Attachment of the levator ani to the rectum. (a) Medial aspect of the female pelvis. LA‐re originated from the Pu (double‐headed arrow line) and ran in the innermost layer of the LA towards the Re. (b) After pulling the Re medially. The LA‐re approached the Re from the anterolateral side and attached to the outer surface of the Re. (c) Magnified view of the rectangular area in b. Smooth muscular tissue extending outward from the LM and attached to the LA‐re (arrows). AC, anal canal; CM, circular muscle (of the rectum); Co, coccygeus; Cx, coccyx; LA, levator ani; LA‐a, LA muscle bundles surrounding the anterior side of the Re; LA‐c, LA muscle bundles attached to the coccyx; LA‐pl, LA muscle bundles laterally surrounding the posterior side of the Re; LA‐re, LA muscle bundles attached to the Re; LM, longitudinal muscle (of the rectum); Mu, mucous membrane (of the rectum); Oi, obturator internus; Pu, pubis; Re, rectum; TALA, tendinous arch of the levator ani.

Observations from the medial aspect showed in more detail how the muscle bundles attached to the rectum (LA‐re). The LA‐re originated from the pubis and ran through the innermost layer of the LA towards the rectum (Figure 2a). To observe the attachment site of the LA‐re to the rectum, the rectum was pulled medially (Figure 2b). The LA‐re approached the rectum from the anterolateral side and the muscle bundles spread anteriorly and posteriorly to attach to the outer surface of the rectal wall. At the attachment site, muscular tissue (specifically smooth muscle) extended outward from the longitudinal muscles of the rectum and attached to the LA‐re (indicated by arrows in Figure 2c).

### 3.2 Sling formation passing anterior to the rectum

**FIGURE 3:**
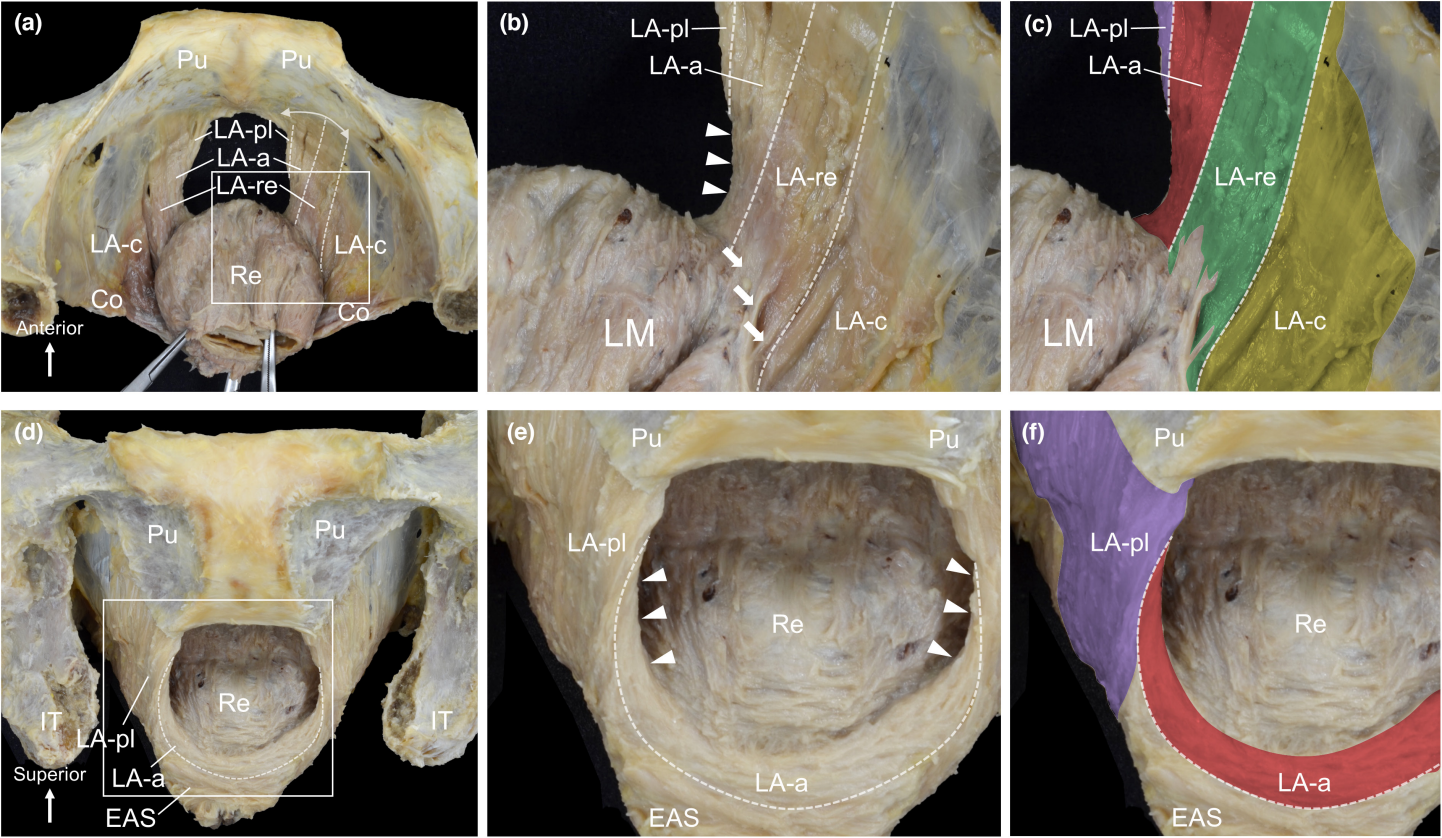
Sling formation passing anterior to the rectum. (a) Superior aspect of the female pelvis. The LA‐a was observed anterior to LA‐re. (b) Magnified view of the rectangular area in a. The smooth muscle tissue extends outward from the LM and attaches to the LA‐re (arrows). Some LA‐a muscle bundles crossed the anterior margin of the LA‐a from the medial to the anterolateral surface (arrowheads). (c) Colour‐coded image of b. (d) Anterior aspect of the same specimen as a. The LA‐a formed a sling that passed anteriorly to the Re. (e) Some LA‐a muscle bundles run from the medial to the anterolateral surface beyond the anterior margin of the LA‐a (arrowheads). (f) Colour‐coded image of e. Co, coccygeus; EAS, external anal sphincter; IT, ischial tuberosity; LA, levator ani; LA‐a, LA muscle bundles surrounding the anterior side of the Re; LA‐c, LA muscle bundles attached to the coccyx; LA‐pl, LA muscle bundles laterally surrounding the posterior side of the Re; LA‐re, LA muscle bundles attached to the Re; LM, longitudinal muscle (of the rectum); Pu, pubis; Re, rectum.

The muscle bundles of the LA were dissected while maintaining their origin (pubic side) and insertion (rectal side). From the superior perspective, muscle bundles attached to the rectum (LA‐re) were observed in the innermost layer, and muscle bundles surrounding the anterior side of the rectum (LA‐a) were observed anterior to the LA‐re (Figure 3a). The LA‐re originated from the pubis and attached to the rectal wall where the muscular tissue (smooth muscle) extended outward from the longitudinal muscle of the rectum and attached to the LA‐re (indicated by the arrows in Figure 3b). LA‐a originated from the pubis and ran towards the anterior side of the rectum alongside some muscle bundles that had been running on the medial surface of the LA‐a, crossing over the anterior margin of the LA‐a and circling to the anterolateral surface (indicated by the arrowheads in Figure 3b). From the anterior aspect, the LA‐a formed a sling that passed anterior to the rectum and, together with the external anal sphincter, constituted the anterior wall of the anal canal (Figure 3d). Some muscle bundles ran from the medial surface to the anterolateral surface beyond the anterior margin of the LA‐a (indicated by the arrowheads in Figure 3e).

### 3.3 Twisted structure of the muscle bundles

**FIGURE 4:**
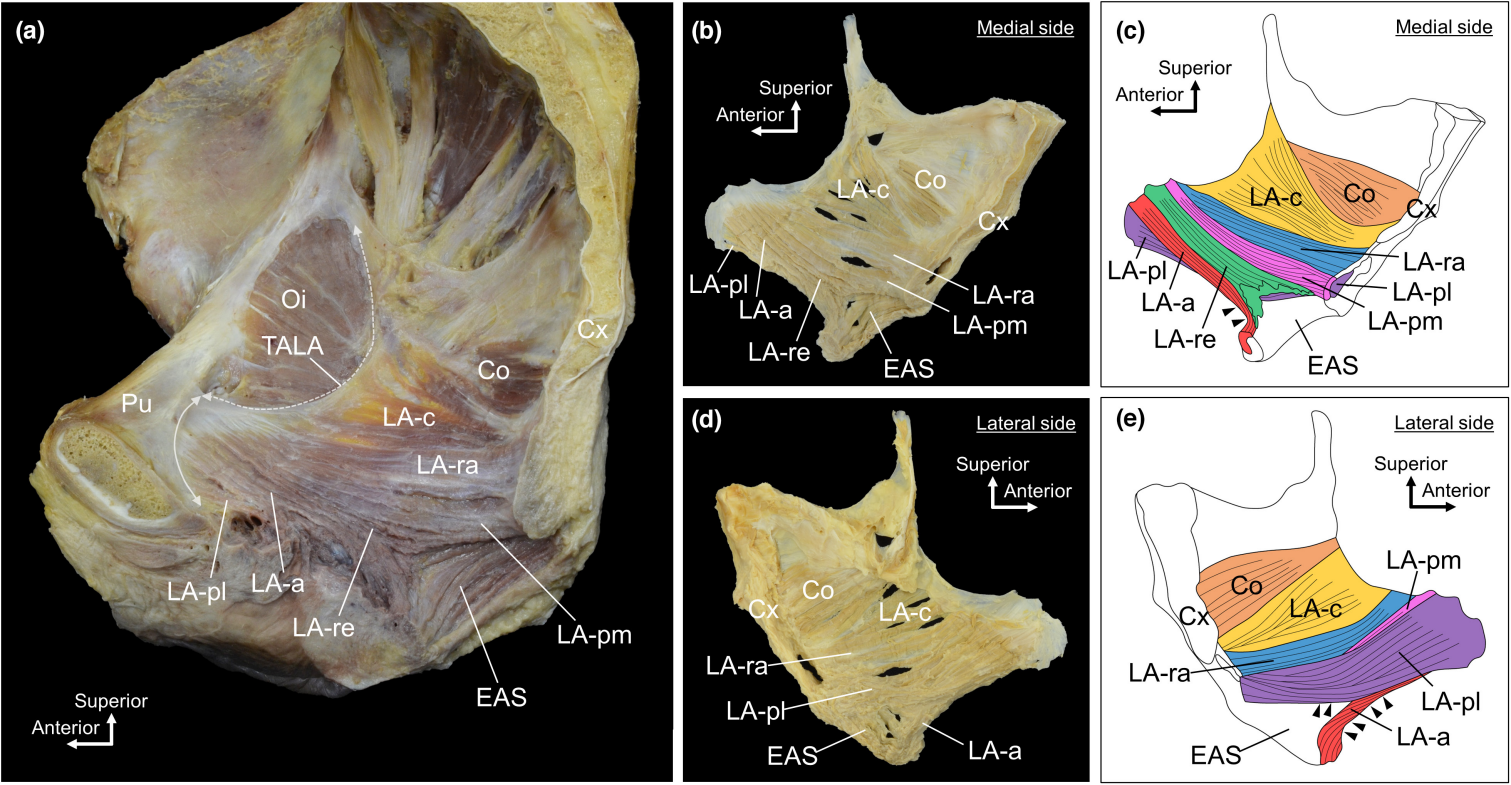
Twisted structure of muscle bundles of the levator ani. (a) Medial aspect of the female pelvis. (b) Medial aspect of the isolated levator ani muscle specimen. The muscle bundles of the LA‐a formed a twisted shape. (c) Colour‐coded image of b. The muscle bundles of the LA‐a, which originate superiorly on the medial surface, run downward and turn outward beyond the anterior margin of the LA‐a (arrowheads). (d) Lateral aspect of the isolated muscle specimen of the levator ani. The muscle bundles of LA‐pl formed a twisted shape. (e) Colour‐coded image d. The muscle bundles of the LA‐pl which originate inferiorly on the lateral surface, run posterosuperiorly. Some muscle bundles running on the medial surface extend beyond the inferior border of the LA‐pl and wrap around the lateral surface (arrowheads). Co, coccygeus; Cx, coccyx; EAS, external anal sphincter; LA, levator ani; LA‐a, LA muscle bundle surrounding the anterior side of the Re; LA‐c, LA muscle bundle attached to the coccyx; LA‐pl, LA muscle bundle laterally surrounding the posterior side of the Re; LA‐pm, LA muscle bundle medially surrounding the posterior side of the Re; LA‐ra, LA muscle bundle attached to the iliococcygeal raphe; LA‐re, LA muscle bundle attached to the rectum; Oi, obturator internus; Pu, pubis; Re, rectum; TALA, tendinous arch of the levator ani.

After dissecting the pelvic half from the medial aspect, the LA was isolated together with the coccygeus and external anal sphincter; the fat and connective tissue on the lateral surface were removed to prepare isolated muscle specimens (Figure 4a,b). The muscle bundles of the LA‐c ran in a fan shape, converging from the TALA at its origin to the coccyx at its insertion. The muscle bundles of the LA‐ra ran almost parallel to each other. The muscle bundles of the LA‐re ran nearly parallel and were slightly widened near the rectum. The muscle bundles of the LA‐a formed a twisted shape, with the muscle bundles originating superior to the medial surface, moving downward and then turning outward beyond the anterior margin of the LA‐a (indicated by the arrowheads in Figure 4c,e). The muscle bundles of the LA‐pl formed a twisted shape in which the muscle bundle started inferior to the lateral surface and turned posterosuperiorly. The twisted shape caused the muscle bundle to run on the medial surface, extend beyond the inferior border of the LA‐pl and wrap around to the lateral surface (indicated by arrowheads in Figure 4e). Both LA‐a and LA‐pl are twisted structures. In both cases, the rotation direction of the twist in the right pelvis was counterclockwise when viewed from the posterior side of the process, from the origin (pubis) to the insertion (surrounding the rectum).

**FIGURE 5:**
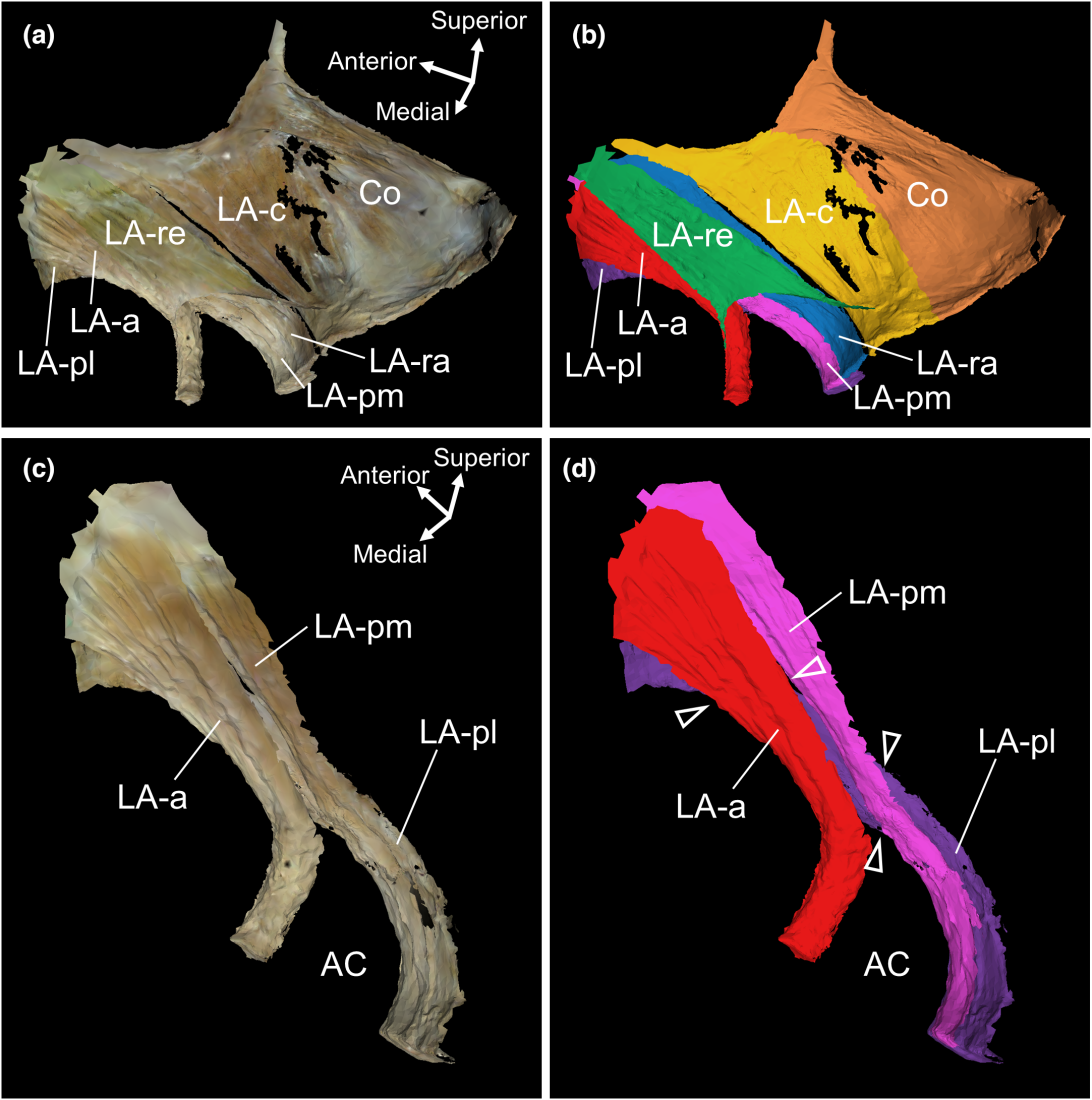
Twisted relationships between muscle bundles of the levator ani. (a) Medial aspects of the LA and Co that were 3D scanned. (b) Colour‐coded image of a. (c) Muscle bundles surrounding the anorectal canal anteriorly and posteriorly. The LA‐pm and LA‐pl and LA‐a and LA‐pl were crossed in their running process (arrowheads) and were in a twisted position with each other. (d) Colour‐coded image of c. AC, anal canal; Co, coccygeus; LA, levator ani; LA‐a, LA muscle bundles surrounding anterior side of the Re; LA‐c, LA muscle bundles attaching to the coccyx; LA‐pl, LA muscle bundles laterally surrounding the posterior side of the Re; LA‐pm, LA muscle bundles medially surrounding the posterior side of the Re; LA‐ra, LA muscle bundles attaching to the iliococcygeal raphe; LA‐re, LA muscle bundles attaching to the Re; Re, rectum.

Based on the 3D scan data of the right pelvic half‐dissected specimen, only portions of the LA and the coccygeus were extracted (Figure 5a,b, Supporting Information S1). In the relationship among LA‐a, LA‐pm and LA‐pl, LA‐a resided in the innermost layer, overlapped with the LA‐pm and LA‐pl at the origin, parted posteriorly from them and then headed toward the anterior side of the rectum (Figure 5c,d). The relationship between the LA‐a and LA‐pl revealed that the former originates from a more superomedial position, whereas the latter originates from a more inferolateral position. Towards the posterior side, they crossed with the LA‐pl, passing outside of the LA‐a towards the posterior rectum (Figure 5d). In terms of the relationship between the LA‐pm and LA‐pl, the LA‐pm originated from a more superomedial position, whereas the LA‐pl originated from a more inferolateral position. In the posterior direction, the two crossed with the LA‐pl passing laterally to the LA‐pm and then curving behind it towards the posterior side of the rectum (Figure 5d). This implies that the muscle bundles surrounding the posterior rectum (LA‐p, LA‐pm and LA‐pl) form a twisted structure, and the LA‐a and LA‐p are arranged in a twisted manner with each other.

## 4 DISCUSSION

**FIGURE 6:**
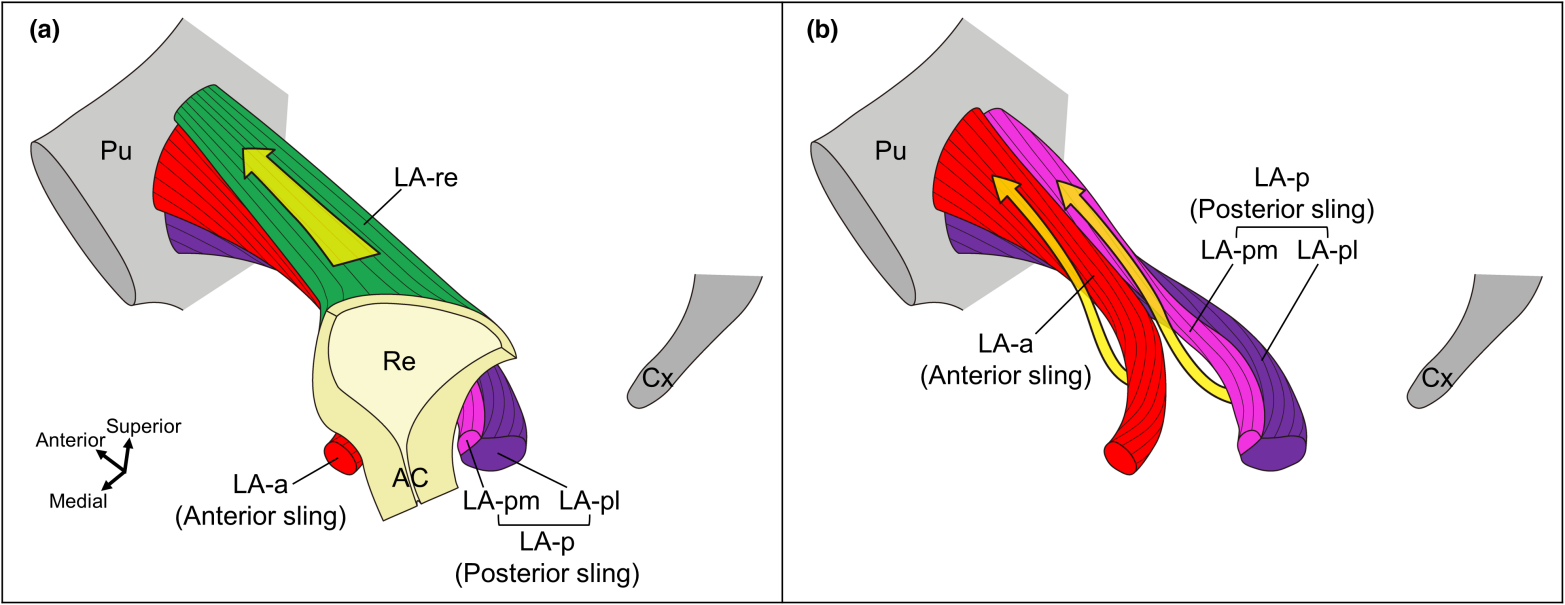
Functional parts of the levator ani. Schemata of the levator ani muscle bundles which anatomically associate with the rectum. (a) Muscle bundles that directly attach to the Re (LA‐re) originating from the pubic bone and inserting into the anterolateral side of the rectal wall. The LA‐re probably contracts to elevate the Re anteriorly and upward, narrowing the levator hiatus and widening the lumen of the Re. (b) Muscle bundles surrounding the anterior and posterior aspects of the Re (LA‐a and LA‐p) exhibit a twisted orientation. LA‐a and LA‐p (anterior and posterior slings) probably work to elevate the Re, anal canal and perineum; narrow the levator hiatus; and narrow the rectal lumen by strengthening the anorectal angle, erecting the anal canal and bringing it upright, by the muscle direction from anterior to posterior and the orientation of their twisted muscle bundles. AC, anal canal; Cx, coccyx; LA, levator ani; LA‐a, LA muscle bundles surrounding anterior side of the Re; LA‐p, LA muscle bundles surrounding the posterior side of the Re; LA‐pl, LA muscle bundles laterally surrounding the posterior side of the Re; LA‐pm, LA muscle bundles medially surrounding the posterior side of the Re; LA‐re, LA muscle bundles attaching to the Re; Pu, pubis; Re, rectum.

This study presents the detailed anatomy, three‐dimensional stratigraphic composition and muscle bundle orientation of the LA. The LA, particularly in its anteroinferior portion, exhibits a complex layered structure comprising overlapping muscle bundles with varying orientations. This portion of the LA contains muscle bundles that are anatomically associated with the rectum. Some muscle bundles directly attach to the rectum, whereas others surround the anterior and posterior aspects of the rectum (Figure 6). All these muscle bundles were found to originate from the pubic bone. Muscle bundles that attach directly to the rectum are mainly inserted into the anterolateral side of the rectal wall. The muscle bundles surrounding the anterior and posterior rectum exhibited a twisted orientation. The portion of the LA directly associated with the rectum is considered the functional portion, as determined by the anatomical characteristics of its origin, insertion and muscle bundle orientation.

Anatomical precision of the pelvic floor holds immense significance in both clinical and research settings (DeLancey, 2022). In particular, the composition or subdivision of the LA remains a contentious issue crucial for clinical diagnosis and treatment. Although the LA is typically divided into three parts, namely the pubococcygeus, puborectalis and iliococcygeus (Standring & Gray, 2015), a systematic review by Kearney et al. (2004) revealed that up to 16 different terms were used in the literature to refer to the five components of the LA. Kearney et al. emphasised the importance of describing each component based on its origin–insertion pair to avoid confusion arising from nomenclature. Specifically, the morphology of LA insertions is complex and variable. Hence, this study describes the results using convenient names that focus on insertions (LA‐re, LA‐a, LA‐pm, LA‐pl, LA‐ra and LA‐c) instead of the usual anatomical terms. The corresponding anatomical terms are listed in Table 1 (Standring & Gray, 2015). The pubococcygeus (pubovisceralis) is sometimes subdivided into several parts according to the pelvic viscera to which each part relates (puboperinealis, puboprostaticus or pubovaginalis, puboanalis); these terms are also found in *Terminologia Anatomica* (Federative Committee on Anatomical Terminology, 1998). The LA‐a in this study corresponds roughly to the puboperinealis and pubovaginalis, and the LA‐re to the puboanalis, but the recognition of morphology differs in the following three points, and it would be inappropriate to assign these conventional anatomical terms as they are. First, the conventional understanding of the puboperinealis is that the LA muscle bundle inserts into the connective tissue anterior to the anal canal (between the rectum and vagina), whereas the muscle bundle of the LA‐a shown in this study does not migrate to tendons or other connective tissue, but is continuous with the contralateral muscle bundle to form a sling structure. Second, the conventional understanding of the pubovaginalis is that the LA muscle bundle inserts into the vaginal wall; however, previous studies have shown that the interface between the LA and the vagina is a different structure with such an understanding (described later). Third, in the conventional understanding of the puboanalis, the LA muscle bundle runs between the internal and external sphincters, but the muscle bundle of the LA‐re shown in this study attaches directly to the longitudinal muscle of the anorectal wall in addition to the intersphincteric run. Therefore, we should be aware of the dissociation between anatomical terminology implying a muscle insertion and the actual anatomical structure, and we should insist on accurate observation and documentation of the structure. Meanwhile, the morphology of the LA origin is also complex and varies between the anterior and posterior portions. The anterior portion of the LA attaches directly to the pubis via the tendons, whereas the posterior portion attaches to the thickened part of the fascia of the obturator internus (obturator fascia), known as the TALA (Kim et al., 2015). Recent studies have shown that the area in which the LA originates from the obturator fascia is broader than previously thought (Muro et al., 2023). However, it remains unclear which parts of the LA originate from the pubis and TALA, as well as the corresponding origin and insertion points.

**TABLE 1:** Terms of the subdivisions of the levator ani and anatomical characteristics.

Terms of the subdivisions of the levator ani					
Present study	LA‐a (anterior sling)	LA‐re	LA‐p (posterior sling)	LA‐ra	LA‐c
*Gray's Anatomy*, 41st ed.	No description	Pubococcygeus (pubovisceralis, puboanalis)	Puborectalis	Pubococcygeus	Iliococcygeus
Anatomical characteristics of the levator ani muscle bundles					
Origin	Pubis (bone)			Tendinous arch of the levator ani	
Insertion	Surrounding the anterior side of rectum (and anal canal)	Wall of rectum (and anal canal)	Surrounding the posterior side of rectum (and anal canal)	Iliococcygeal raphe	Coccyx
Muscle bundle orientation	Twisted shape	Parallel, widened in inversion	Twisted shape	Parallel	Fan shape

*Note*: LA, levator ani; LA‐a, LA muscle bundle surrounding the anterior side of the rectum; LA‐c, LA muscle bundle attached to the coccyx; LA‐p, LA muscle bundle surrounding the posterior side of the rectum; LA‐ra, LA muscle bundle attached to the iliococcygeal raphe; LA‐re, LA muscle bundle attached to the rectum.

Several reports have been published regarding the composition of LA. While previous detailed gross anatomical methods allowed for detailed observation of the muscles, the primary data were recorded as photographs or sketches, making visualisation in three dimensions difficult (Ayoub, 1979; Baramee et al., 2020; Courtney, 1950; Shafik, 1975; Smith, 1923; Suriyut et al., 2020; Thompson, 1899). However, modern imaging techniques such as magnetic resonance imaging (MRI), computed tomography (CT), 3D ultrasound and sectional anatomy by the Visible Human Project have enabled 3D rendering and reconstruction for the analysis of LA composition in three dimensions (Guo & Li, 2007; Li et al., 2015; Manzini et al., 2021; Wu et al., 2015, 2017). Nevertheless, the cross‐sectional images used in the 3D analyses have limitations in revealing information regarding the orientation of muscle bundles. Alternatively, studies using diffusion tensor imaging (DTI) have enabled the visualisation of muscle bundle orientation, reporting differences in the orientation and angle between the pubovisceralis (pubococcygeus) and the puborectalis of the LA, but have only analysed the sagittal plane in two dimensions (Betschart et al., 2014). A study using photogrammetry also visualised the 3D arrangement of muscle bundles, but it only analysed the muscle bundles on the surface of the LA (Routzong et al., 2021). The present study comprehensively clarified the anatomy of LA muscle bundles, including their 3D stratigraphic composition, orientation and the correspondence between the origin and insertion. One of the most detailed previous studies describing the LA composition is by Lawson (1974), who presents illustrations showing the detailed muscle composition. Lawson's study was based on serial sections of neonatal and infant specimens. In contrast, our present study is significant in that we performed the precise macroscopic dissection using adult cadavers to display the specific fibre directions of component parts of the LA. Ayoub (1979) noted that the anterior portion of the LA has a twisted structure; however, anatomical information is lacking and there is no description of the twist orientation or muscle bundle arrangement. To the best of our knowledge, this is the first detailed report of the twisted shape of the LA muscle bundles that surround the rectum anteriorly and posteriorly (LA‐a and LA‐p: anterior and posterior slings).

The parts of the LA involved in the rectum work to elevate the rectum and anal canal (hence named ‘levator ani’) and are responsible for pelvic floor support and urinary and defaecatory functions. Among the LA muscle bundle configurations identified in this study, those involved in the rectum were LA‐re, which was attached to the rectum, and LA‐a and LA‐p (LA‐pm and LA‐pl), which surrounded the rectum anteriorly and posteriorly respectively. All of these muscle bundles originate from the pubic bone, which provides a more mechanically stable fulcrum than that originating from the TALA. Moreover, these muscle bundles are thicker and more multilayered than the rest of the LA (LA‐ra and LA‐c) and are likely the primary areas in the LA that exert contractile force. Based on these findings, the LA‐re, LA‐a and LA‐p can be identified as the functional parts of the LA.

The morphology of the functional parts of the LA provides important insights into muscle function. The insertion of the LA‐re does not form connective tissue tendons or aponeurosis; rather, skeletal muscle fibres attach directly to the smooth muscle fibres of the rectal wall (Muro et al., 2020; Tsukada et al., 2016). It is widely attached to the lateral wall of the rectum, particularly to the anterolateral facet. The LA‐re likely contracts to elevate the rectum anteriorly and upward, narrowing the levator hiatus and widening the lumen of the rectum (Figure 6a). Conversely, the LA‐a and LA‐p, which surround the rectum anteriorly and posteriorly, form a twisted structure. Considering the direction of the twisting rotation, the rotational force (torsional moment = torque) generated during muscle contraction seems to raise the anteriorly inclined anal canal to bring it upright. In other words, it elevates the centre of the perineum (the region anterior to the rectum). Therefore, the LA‐a and LA‐p likely work to elevate the rectum, anal canal and perineum; narrow the levator hiatus; and narrow the rectal lumen by strengthening the anorectal angle, erecting the anal canal and bringing it upright, by the muscle direction from anterior to posterior and the orientation of their twisted muscle bundles (Figure 6b). These muscle bundles appear to be closely associated with the pathogenesis of perineal descent (Brillantino et al., 2023; Chaudhry & Tarnay, 2016; Wang et al., 2020). A study that used video myogram defaecography and MRI defaecating proctogram reported that the activity of the puborectalis (corresponding to the LA‐p in this study) is important for the defaecation process (Petros et al., 2012).

In addition, the LA‐a is near the lateral aspect of the vagina and is therefore considered to be attached to the vagina. A part of the LA is known to attach to the lateral aspect of the vagina, and this structure is believed to influence the proximal position of the urethra (DeLancey & Starr, 1990). This connection between the LA and the vagina is not a result of LA muscle fibres inserting into the vaginal wall, but rather smooth muscle fibres extending from the vaginal wall interlocking with the medial surface of the LA skeletal muscle bundles to form a solid interface: skeletal muscle sandwiched by smooth muscle and smooth muscle inserting into skeletal muscle (DeLancey & Starr, 1990; Kato et al., 2020; Muro & Akita, 2023b). Therefore, the LA‐a may also contribute to the support and elevation of the vagina and stabilisation of the proximal urethral position. These anatomic features of the LA‐a provide important insights into birth‐related LA muscle injuries. The LA‐a, which forms a sling posterior to the vagina, is probably directly and significantly stretched and vulnerable to injury during delivery. A study of 3D structural model simulation has shown that the part of LA that stretches the most during vaginal delivery is the most medial and ventral part, which corresponds to the LA‐a shown in our present study (Lien et al., 2004). Therefore, the LA‐a should be considered a site of concern for injury during vaginal delivery. Presumably, a portion of the LA‐a sling is palpable for the obstetrician/gynaecologist on examination. During physical examination, the tissue between the anal canal and the vagina can be palpated. This tissue is commonly recognised as the ‘central tendon’ or ‘perineal body’, but this palpable tissue contains the LA‐a muscle bundle.

Our study has a few limitations. First, the ages of the subjects were skewed owing to the use of cadavers for elderly adults, who averaged >80 years old. Second, comparison of the findings of this study with vaginal delivery history was not possible because no relevant data on delivery history were collected during the body donation process. Third, the shape of the muscle may be distorted compared with the living bodies. In future studies, comparison of the shapes observed in cadavers with MRI images of living bodies is needed. Fourth, this study did not histologically evaluate the origin or insertion of the LA. Finally, this study was purely anatomical; therefore, quantitative measurements of the LA movement were not possible. Future biomechanical research may offer additional insight into the function of each LA component, making the comprehensive anatomical findings of this study useful.

## 5 CONCLUSION

This study clarified the stratigraphic composition, orientation and correspondence between the origin and insertion of the LA. Based on detailed morphology, the functional parts of the LA were identified, which consisted of muscle bundles directly attached to the rectum and muscle slings surrounding the rectum anteriorly and posteriorly. The twisted structures of the anterior and posterior slings of the LA indicate that these slings possess a distinctive muscle bundle orientation that is crucial for functional performance. These findings provide a significant anatomical foundation for the exploration of pelvic floor support mechanisms, pathogenesis of pelvic floor disease and accurate diagnosis and treatment.

## INSTITUTIONAL REVIEW BOARD APPROVAL

The Board of Ethics at Tokyo Medical and Dental University approved the study (approval number: M2018‐006). All methods were performed following the relevant guidelines and regulations.

## AUTHOR CONTRIBUTIONS

Satoru Muro contributed to the conception and design of the work; acquisition, analysis interpretation of data; drafting of the manuscript; and final approval of the version to be published. Shoko Moue contributed to the acquisition, analysis and interpretation of data; critical revision of the draft; and final approval of the version to be published. Keiichi Akita contributed to the conception and design of the work, interpretation of data, critical revision of the draft and final approval of the version to be published.

## FUNDING INFORMATION

This study was supported by JSPS KAKENHI (grant numbers JP19K23821, JP21K15329 and JP21H03799).

## CONFLICT OF INTEREST STATEMENT

The authors declare no conflicts of interest to declare, financial or otherwise.

## Supporting information


Appendix S1:

## Data Availability

Data supporting this study's findings are available from the corresponding author on a reasonable request.
