# Supplementary figures and images for: Twisted orientation of the muscle bundles in the levator ani functional parts in women: Implications for pelvic floor support mechanism
Source: J Anat. 2023 Oct 26;244(3):486–96. doi: 10.1111/joa.13968 (PMC10862148; doi:10.1111/joa.13968)

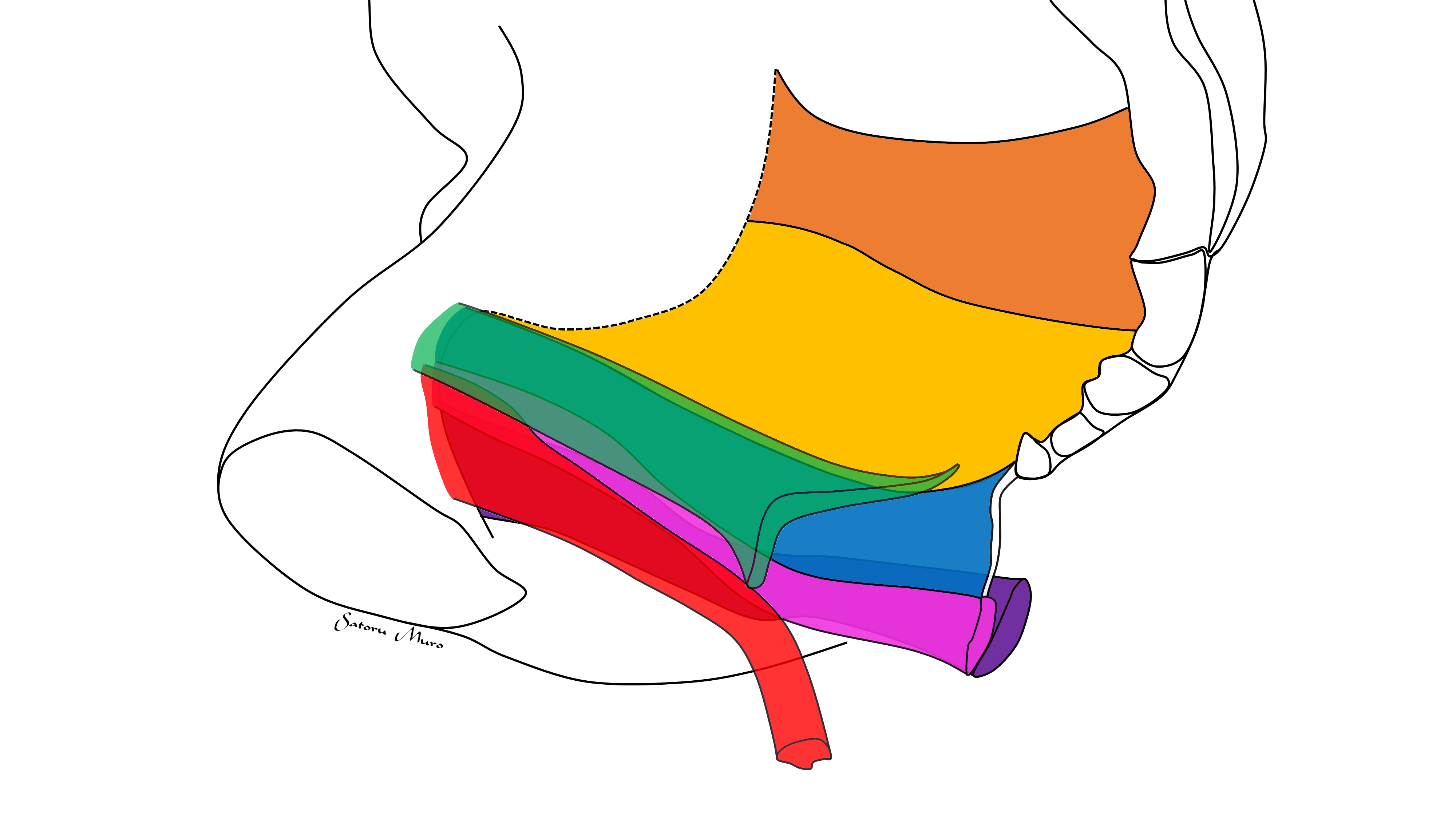

Supplement: Supplementary file 1 — Appendix S1: [file JOA-244-486-s001.zip › Illustration1.png]

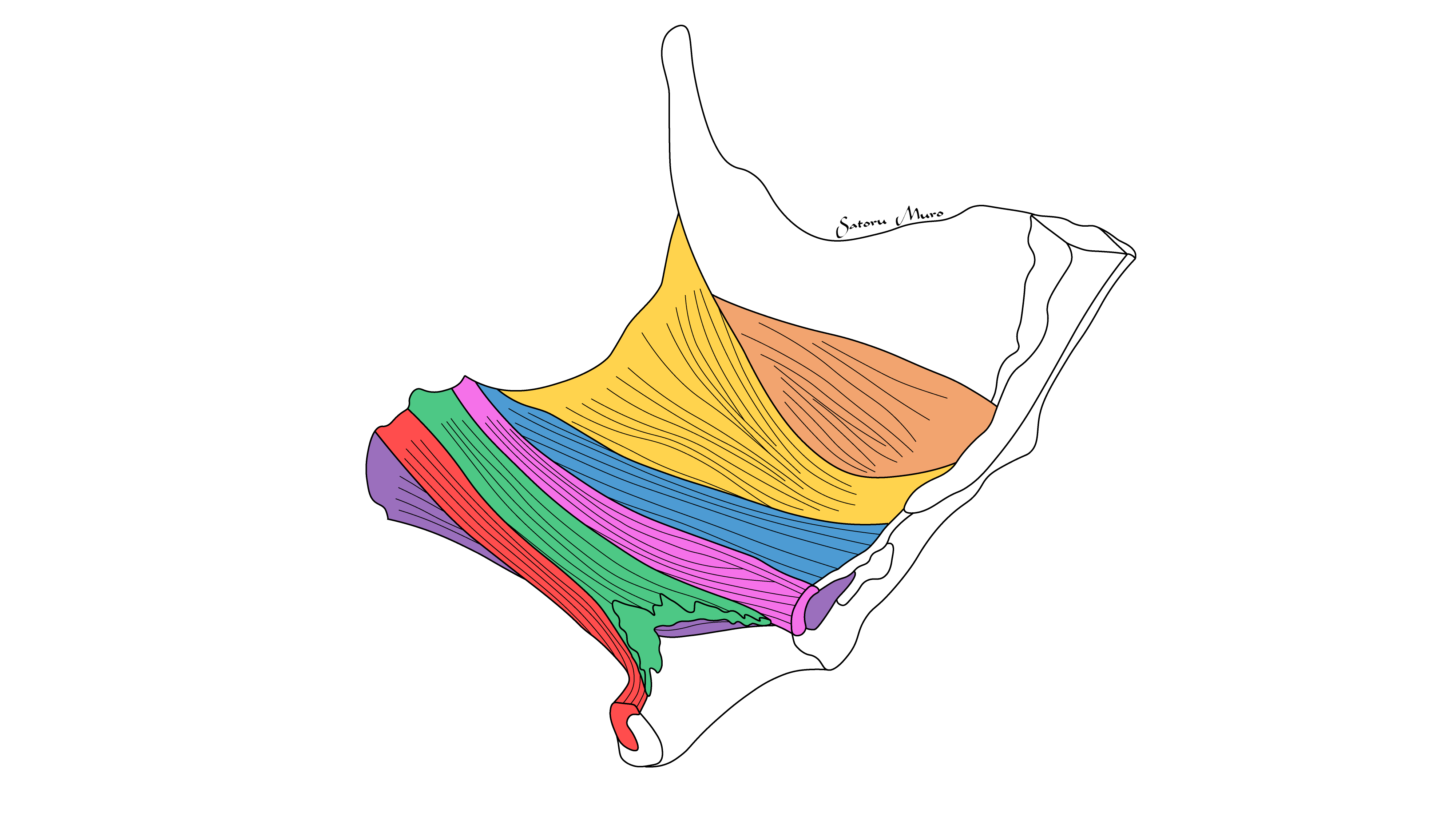

Supplement: Supplementary file 1 — Appendix S1: [file JOA-244-486-s001.zip › Illustration2.png]

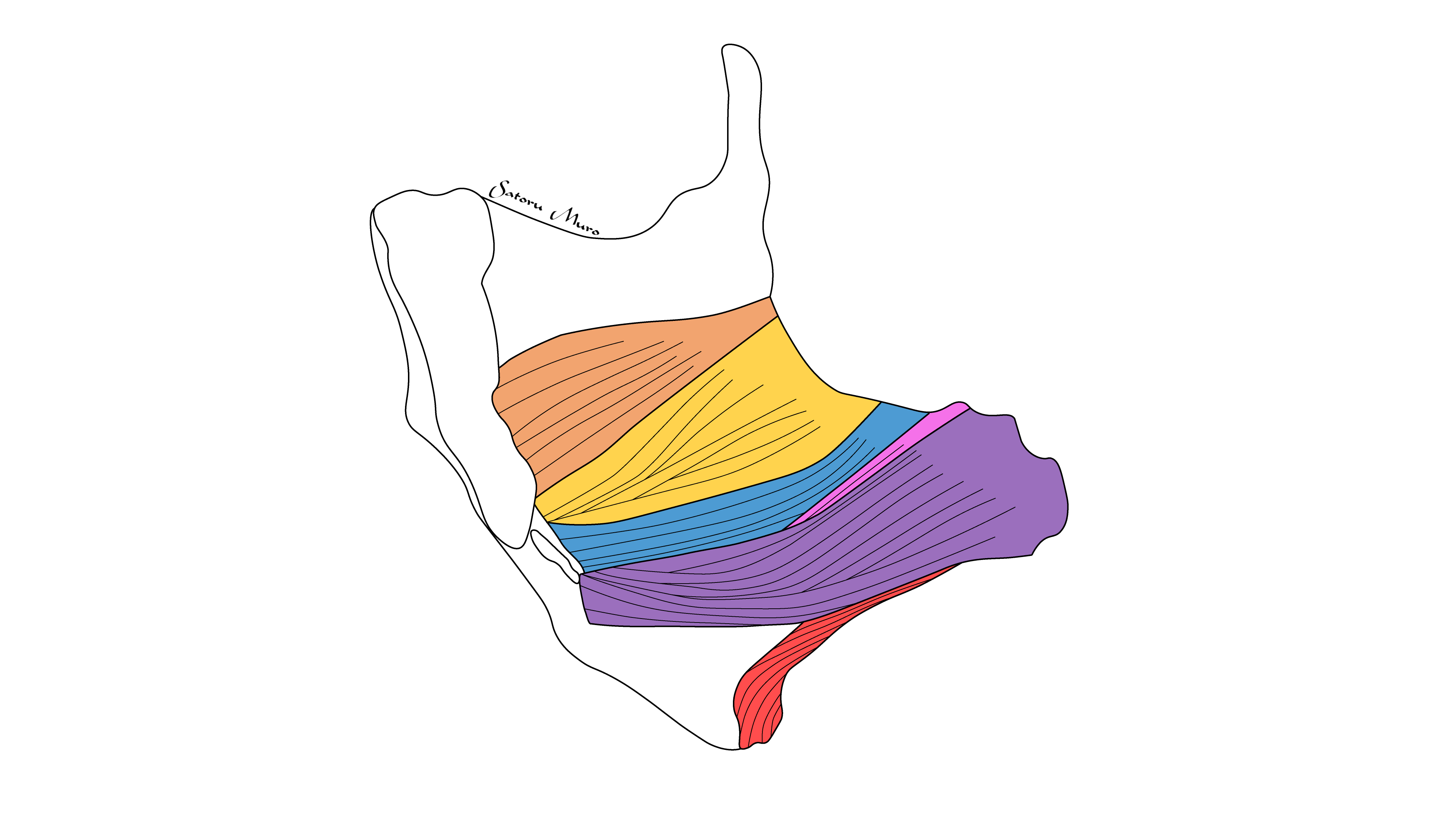

Supplement: Supplementary file 1 — Appendix S1: [file JOA-244-486-s001.zip › Illustration3.png]

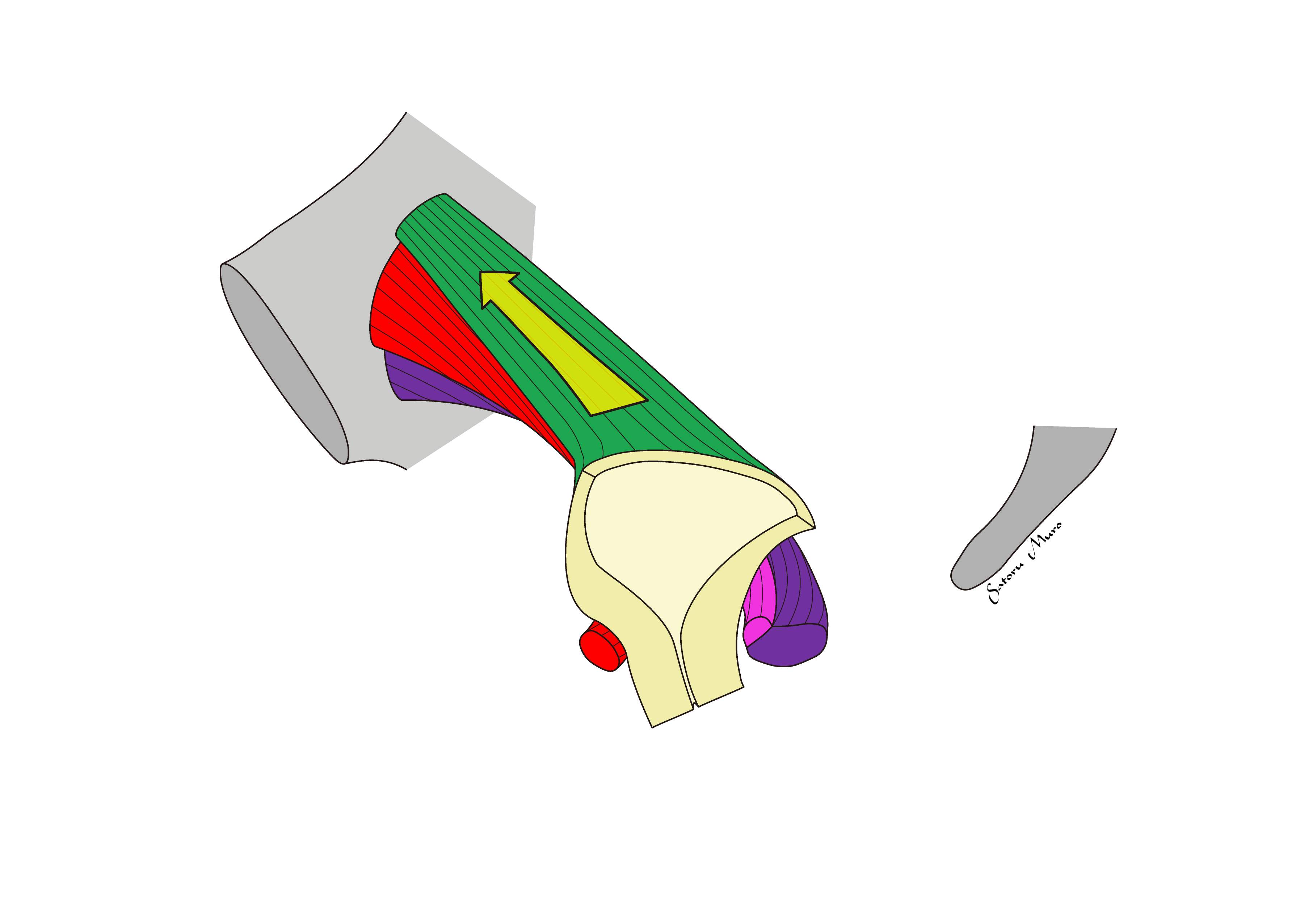

Supplement: Supplementary file 1 — Appendix S1: [file JOA-244-486-s001.zip › Illustration4.png]

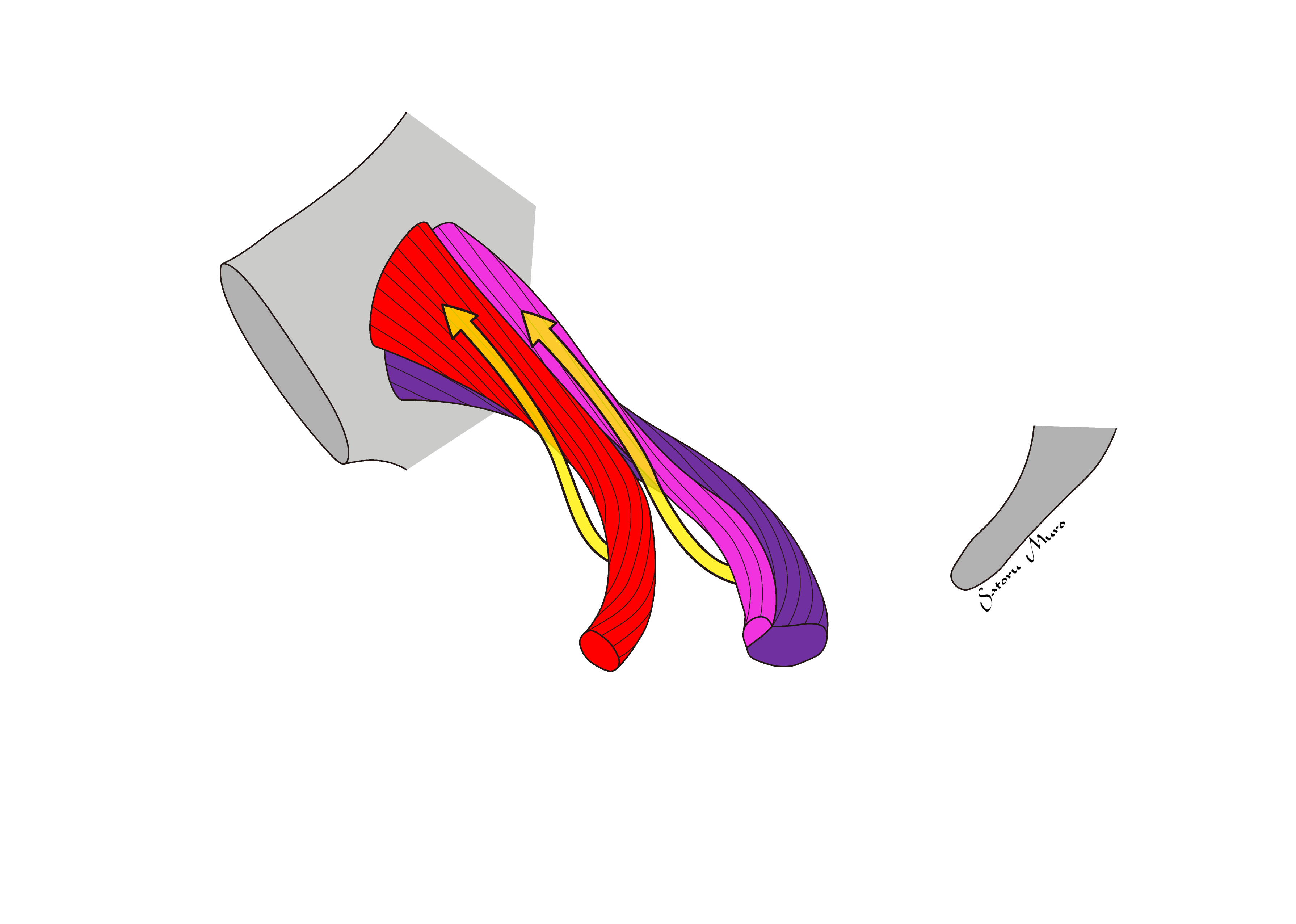

Supplement: Supplementary file 1 — Appendix S1: [file JOA-244-486-s001.zip › Illustration5.png]
